# High-Precision Tunneling Magnetoresistance (TMR) Current Sensor for Weak Current Measurement in Smart Grid Applications

**DOI:** 10.3390/mi16020136

**Published:** 2025-01-24

**Authors:** Yong Xu, Zhenhu Jin, Jiamin Chen

**Affiliations:** 1 State Key Laboratory of Transducer Technology, Aerospace Information Research Institute, Chinese Academy of Sciences, Beijing 100190, China; xuyong22@mails.ucas.ac.cn; 2 School of Electronic, Electrical and Communication Engineering, University of Chinese Academy of Sciences, Beijing 100049, China; 3 College of Materials Sciences and Opto-Electronic Technology, University of Chinese Academy of Sciences, Beijing 100049, China

**Keywords:** tunneling magnetoresistance current sensor, smart grid, leakage current detection, non-contact

## Abstract

To meet the demand for high-precision, high-resolution measurement of weak currents in smart grids, this article presents the design of a current sensor based on the tunneling magnetoresistance (TMR) effect. To improve the detection accuracy of the sensor, this design adopts a low-noise, high-sensitivity TMR chip as its chip selection; in the sensor circuit, a high-linearity interface circuit is used to eliminate fixed bias; and a magnetic flux concentrator is used to improve sensitivity and anti-interference capability. Experimental results indicate that the sensor achieves a sensitivity of 29.4 mV/V/mA, a linearity of 0.19%, and an accuracy of 0.045% within a ±100 mA range, supporting current measurement from DC up to 10.5 kHz. The proposed sensor demonstrates several advantages, including a wide measurement range, high accuracy, high resolution, and non-invasive measurement capability, making it well suited for weak current detection in smart grid applications.

## 1. Introduction

The development of smart grids is trending toward greater safety, sustainability, and efficiency [1,2,3]. Accurate and real-time current measurement is essential for the monitoring, analysis, and decision-making processes that ensure the stable operation of the power system [4]. In smart grids, weak current monitoring plays a crucial role, particularly in scenarios such as monitoring line insulators, lightning arrester leakage currents, and transformer core ground currents. These currents are typically small in amplitude, ranging from several hundred microamperes to several tens of milliamperes [5]. Among these applications, lightning arresters serve as vital protection devices for electrical equipment, power lines, and personnel. They prevent overvoltage damage caused by lightning strikes, grid mutations, and load fluctuations by quickly conducting and directing excess voltage into the ground, thereby safeguarding the system’s integrity. The leakage current from these arresters can be used to assess the degree of aging and overall health of these devices [6].

As smart grids evolve, there is an increasing demand for current sensors that offer higher accuracy, resolution, and measurement range [7,8]. Compared with other magnetic sensors, TMR current sensors have a wide linear measurement range, high resolution, high sensitivity, and low power consumption, which is very suitable to meet these new requirements [9]. Thus, the detection of weak currents is of critical importance in the context of advanced smart grid technologies. Table 1 is a performance comparison of magnetic sensors.

This study presents the design of a non-contact micro-current sensor based on the TMR effect, aimed at addressing the growing need for high-precision, non-invasive current measurement in smart grids. The sensor utilizes a magnetic flux concentrator (MFC) to enhance both sensitivity and anti-interference performance, and incorporates three TMR chips to minimize measurement errors caused by wire eccentricity. A test system was developed to characterize the sensor’s sensitivity, accuracy, and resolution. The primary factors limiting measurement accuracy were also analyzed. Experimental results demonstrate that the proposed sensor offers high precision and resolution, making it a viable solution for weak current measurement in smart grid applications.

## 2. Experimental Principle

**Figure 1:**
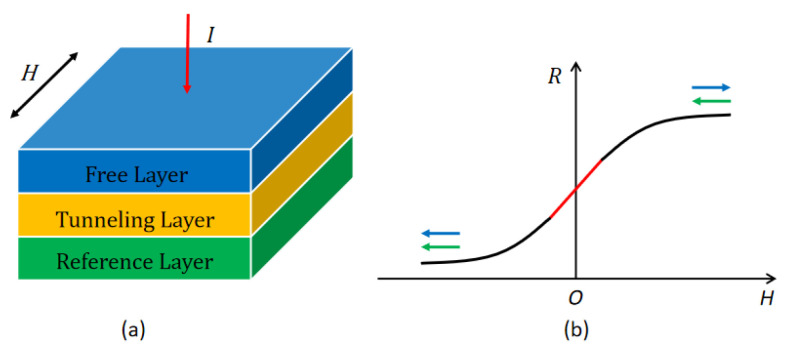
Working principle of TMR chip: (**a**) layer structure of magnetic tunnel junction, and (**b**) R–H curve of magnetic tunnel junction.

The magnetic tunnel junction (MTJ) is the basic magnetic sensing unit of the TMR chip. Each MTJ is composed of three layers: a reference layer, a tunnel barrier layer, and a free layer. The structure of the magnetic tunnel junction is depicted in Figure 1a, where *H* represents the applied magnetic field, *I* is the current passing through the device, and *R* denotes the resistance of the MTJ. The resistance of the MTJ depends on the relative magnetization orientations of the free and reference layers. As the applied magnetic field changes, the magnetization directions of these layers also change, which in turn affects the resistance [14]. The resistance–magnetic field (R–H) characteristic of the MTJ is shown in Figure 1b. When the magnetization directions of the free and reference layers are aligned, resistance is at a minimum. Conversely, when the magnetization directions are opposite, resistance reaches its maximum. Within a specific range of applied magnetic fields, the resistance of the MTJ exhibits a proportional relationship to the strength of the applied magnetic field.

**Figure 2:**
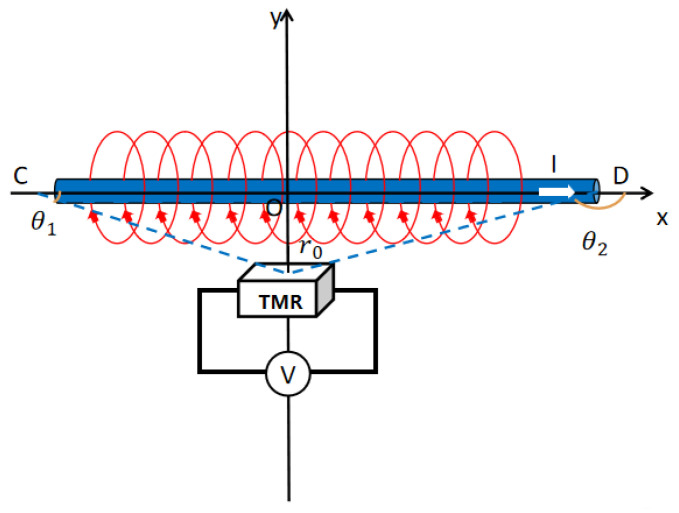
Schematic diagram of TMR sensor measuring current.

When a current flows through a conductor, it generates a magnetic field surrounding the conductor. The TMR chip detects this magnetic field and converts the magnetic signal into a measurable voltage. The magnitude of the measured voltage correlates with the strength of the magnetic field, which can be used to determine the current flowing through the conductor. The schematic diagram of the TMR current sensor, as shown in Figure 2, illustrates this principle of operation.

According to the Biot–Savart law, the magnetic field B at a point near a current-carrying conductor can be expressed as:

(1) $$B=\frac{\mu_{0}I}{4\pi r_{0}}\int_{\theta_{1}}^{\theta_{2}}\sin\theta d\theta =\frac{\mu_{0}I}{4\pi r_{0}}(\cos\theta_{1}-\cos\theta_{2})$$

where μ_0_ is the permeability of free space (4π × 10^−7  ^N/A^2^), I is the current flowing through the conductor, r_0_ is the perpendicular distance from the TMR chip to the conductor, and *θ* is the angle between the line connecting the TMR chip to a point on the conductor and the positive *x*-axis. For an infinitely long conductor, the magnetic field B at the location of the TMR chip simplifies to:

(2) $$B=\frac{\mu_{0}I}{2\pi r_{0}}$$

From this relationship, it is evident that the magnetic field strength is directly proportional to the current flowing through the conductor. Consequently, the differential output voltage of the TMR chip, which is proportional to the magnetic field strength, can be used to measure the current. By accurately measuring the output voltage, the magnitude of the current can be determined.

## 3. Sensor Structure and Design

### 3.1. Sensor Structure Design

**Figure 3:**
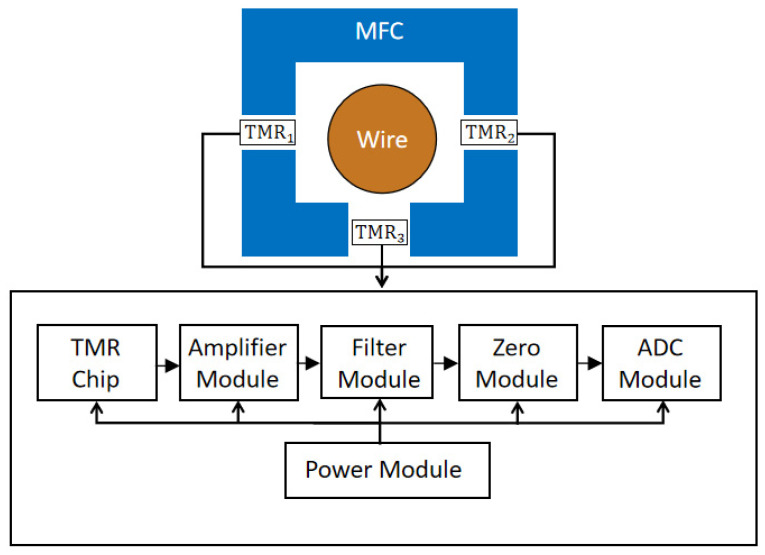
Schematic diagram of the sensor structure.

**Figure 4:**
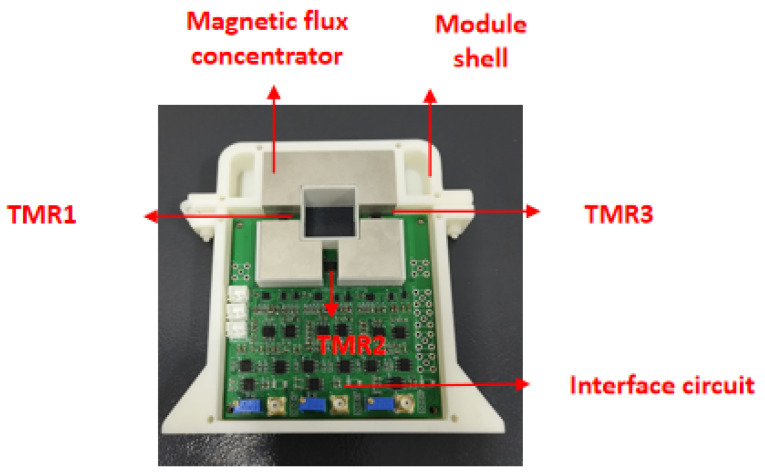
Physical diagram of the sensor.

The sensor system consists of three main components: a module shell, a magnetic flux concentrator, and a sensor interface circuit. The schematic diagram and physical layout of the sensor are illustrated in Figure 3 and Figure 4, respectively. The TMR chip is positioned within the three-gap magnetic flux concentrator, where the differential voltage is generated by measuring the magnetic field produced by the current-carrying conductor within the gap. The sensor interface circuit comprises several key modules, including a power supply, an amplifier, a filtering module, and a zero module. The power supply module provides the necessary power for the entire sensor circuit. The amplifier module amplifies the weak output signal from the sensor to a suitable level for further processing. The filtering module removes unwanted noise and interference from the amplified sensor signal, improving the signal–noise ratio. The zero module compensates for any DC offset or zero-point drift in the sensor output, ensuring accurate measurement. The ADC module converts the analog output signal from the sensor into a digital signal. The coordinated work of these functional modules is crucial for the sensor interface circuit to effectively condition and process the sensor’s output, optimizing the overall performance of the sensing system.

**Figure 5:**
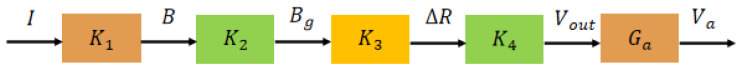
Mathematical model of the TMR current sensor.

In this study’s sensor design, the measured physical quantity (current) is first converted into a magnetic field. The magnetic field then induces a change in the magnetoresistance of the TMR chip, which in turn results in a corresponding change in the output voltage. This output voltage is subsequently amplified by an operational amplifier for further processing. To model and analyze the sensor system, a transfer function block diagram has been established, as shown in Figure 5.

In Figure 5, I represents the measured conductor current; $K_{1}$ represents the conversion coefficient from current to magnetic field; B represents the magnitude of the magnetic field generated by the conductor in the gap; $K_{2}$ represents the amplification factor of the magnetic flux concentrator; $B_{g}$ represents the magnetic field after amplification by the magnetic flux concentrator; $K_{3}$ represents magnetoresistance sensitivity; ΔR represents the change in magnetoresistance; $K_{4}$ represents the sensitivity of the TMR sensor chip; $V_{out}$ represents the output voltage of the TMR sensor chip; $G_{a}$ represents the amplification factor of the sensor interface circuit, which is mainly determined by the gain of the operational amplifier; and $V_{a}$ represents the output voltage of the operational amplifier in the sensor interface circuit.

These factors contribute to the overall performance and sensitivity of the sensor, allowing the system to accurately measure weak currents in a variety of applications.

### 3.2. TMR Chip Selection

To improve the detection accuracy of weak electrical currents, it is crucial to select TMR chips with high sensitivity and low noise characteristics. The TMR current sensor utilizes the TMR chip as a basic magnetic field sensing unit. This chip employs a Wheatstone full-bridge structure and offers a sensitivity of 100 mV/V/Gs, with a background noise of 250 pT/√Hz at 1 Hz. Furthermore, the chip features low hysteresis and low power consumption, making it well suited for applications that require high precision and low energy consumption.

### 3.3. Magnetic Flux Concentrator Design

To minimize measurement errors and enhance the sensor’s sensitivity, a three-gap magnetic flux concentrator is utilized. The use of three TMR chips allows the measurement of the current to be averaged, thus reducing errors from potential eccentricities or misalignments in the sensor setup [15]. To optimize the magnetic flux concentrator effect, the gap spacing of the concentrator should be as small as possible [16].

**Figure 6:**
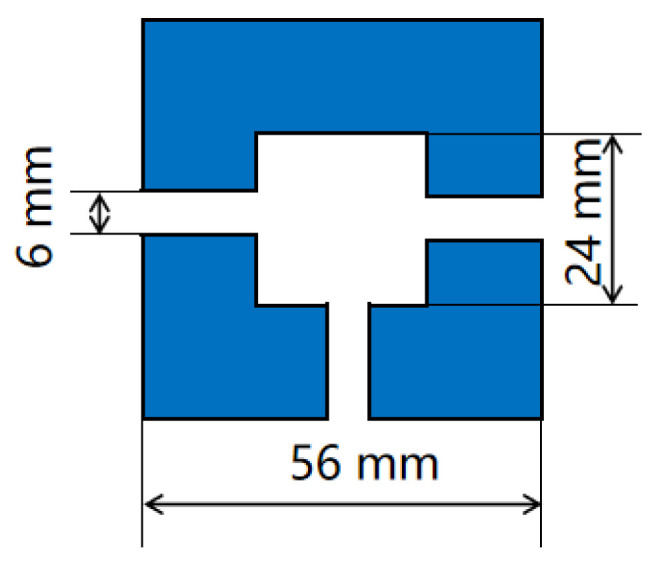
Dimensional schematic of the magnetic flux concentrator.

The magnetic flux concentrator was designed with the following specifications: a gap spacing of 6 mm, an inner diameter of 24 mm, an outer diameter of 56 mm, and a thickness of 17 mm. The dimensional schematic of the magnetic flux concentrator is shown in Figure 6. Permalloy, a material with low hysteresis, high permeability, and small coercivity, was chosen for the concentrator due to its excellent magnetic properties, which help enhance the overall performance of the sensor.

## 4. Experiments and Discussion

### 4.1. Static Characteristics Experiment Regarding the Sensor

**Figure 7:**
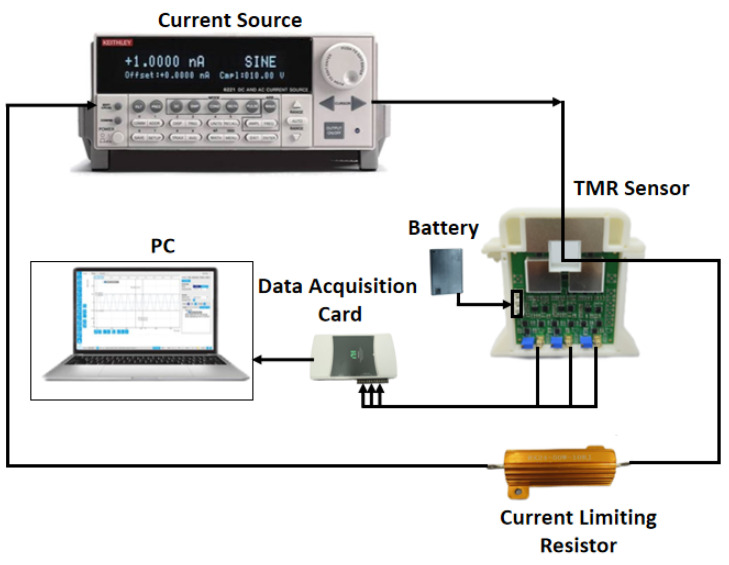
Static characteristic test system.

The static characteristics of the sensor, including sensitivity, linearity, and accuracy, are critical for assessing its performance in real-world applications [17]. The experimental setup used for testing the static characteristics of the sensor is depicted in Figure 7. A Keithley 6221 current source (Tektronix Inc., Beaverton, OR, USA) is employed to provide a stable current input. To account for potential load effects on the conductor loop, a 10 Ω, 10 W RX24 current-limiting resistor is connected in series with the output of the current source. The TMR sensor is powered by a battery, and a data acquisition card is used to interface with the sensor’s output, transmitting data to a computer for analysis. The sensor is enclosed within a shielding tube during testing to minimize external environmental interference. Experiments were all conducted at normal room temperature and humidity.

#### 4.1.1. Range Test

**Figure 8:**
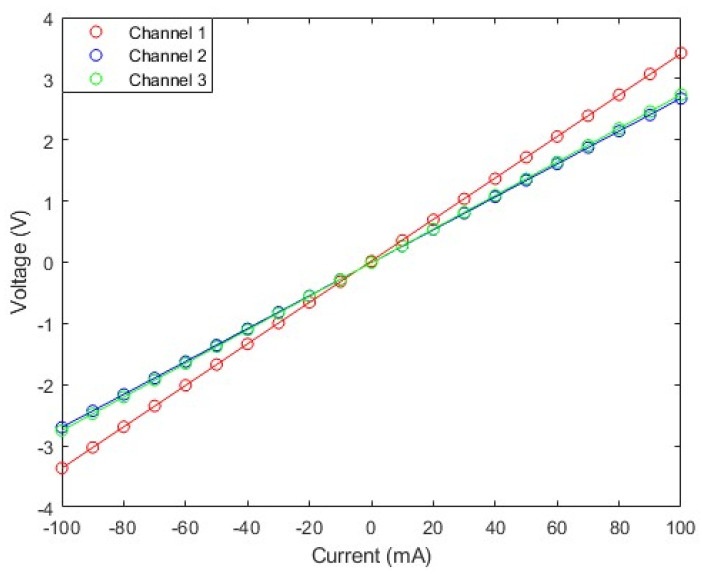
Input–output characteristic curve.

The measurement range of the TMR current sensor was first determined by calibrating the sensor’s DC input–output characteristic curve using the static characteristic test system. The resulting input–output characteristic curve is shown in Figure 8. The measurement range of the sensor is defined as the algebraic difference between the maximum and minimum measurable current values. Based on this test, the TMR current sensor was found to have a measurement range of 200 mA.

#### 4.1.2. Sensitivity Test

The TMR magnetic sensor chip, as a resistive component in a bridge structure, has an output voltage related to the supply voltage of the chip. Therefore, the sensitivity of the TMR current sensor is defined as the ratio of the change in the sensor output voltage to the product of the corresponding change in the input current and the supply voltage, which can be represented by the slope of the best-fit line of the sensor’s input–output curve divided by the supply voltage. By using the polyfit polynomial fitting function in Matlab 2023b to fit test data, the linear fitting lines of the input–output characteristic curves of the three channels are obtained as follows:

(3) $$y_{1}=0.0339x_{1}+0.0216$$

(4) $$y_{2}=0.0269x_{2}-0.0086$$

(5) $$y_{3}=0.0274x_{3}-0.0071$$

Therefore, the slopes of the linear fitting lines of the three channels of the TMR current sensor are 33.9 mV/mA, 26.9 mV/mA, and 27.4 mV/mA, respectively, with the TMR chip supply voltage being 1 V. The sensitivities are 33.9 mV/V/mA, 26.9 mV/V/mA, and 27.4 mV/V/mA, respectively, and the average sensitivity of the current sensor is 29.4 mV/V/mA.

#### 4.1.3. Linearity Analysis

Linearity represents the degree of consistency between the test curve and the fitting line, referring to the maximum deviation of the average input–output characteristic curve of the forward and reverse strokes of the sensor relative to the fitting line, expressed as a percentage of the full-scale output:

(6) $$\delta_{L}=\frac{\Delta U_{max}}{U_{FS}}\times 100\%$$

where $\delta_{L}$ is the linearity of the sensor; $\Delta U_{max}$ is the absolute value of the maximum difference between the arithmetic mean of the output voltages measured multiple times at the same input current in the forward and reverse strokes, and the corresponding point on the fitting line; and $U_{FS}$ is the full-scale output. Calculating the absolute value of the linearity deviation of the TMR current sensor in the ±100 mA range, and taking the maximum deviation divided by the full-scale output voltage, the linearities of the TMR current sensor in the ±100 mA range are 0.19% FS, 0.09% FS, and 0.06% FS. The smaller the linearity, the better the linear fitting of the sensor is. It can be seen that the TMR current sensor has very good linearity.

#### 4.1.4. Accuracy Analysis

**Figure 9:**
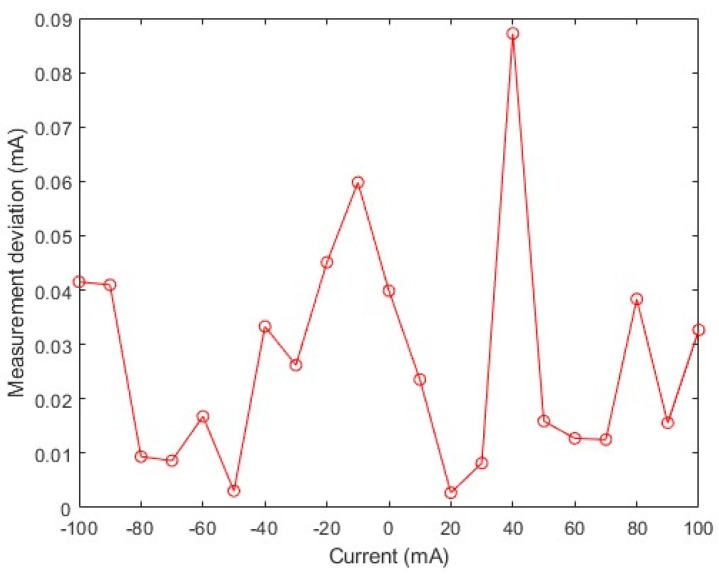
Sensor measurement deviation.

The accuracy of the TMR current sensor refers to the degree of consistency between the measurement result and the true value of the input current, expressed as the maximum deviation between the measured value of the input current and its true value, as a percentage of the full scale of the TMR current sensor:

(7) $$\delta_{A}=\frac{\Delta I_{max}}{I_{FS}}\times 100\%$$

where $\delta_{A}$ is the accuracy of the sensor; $\Delta I_{max}$ is the absolute value of the maximum difference between the arithmetic mean of the input current measurement values measured multiple times at the same input current in the forward and reverse strokes and the true value of the input current, where the true value of the input current is recorded as the output setting value of the low-noise current source; and $I_{FS}$ is the full scale of the current. Figure 9 shows the absolute value of the measurement deviation of the TMR current sensor in the ±100 mA range.

Based on the sensor measurement deviation, the maximum absolute value of the measurement deviation of the TMR current sensor within the ±100 mA range is 0.09 mA, and according to Formula (7), the accuracy of the TMR current sensor is 0.045%. The closer the accuracy value of the TMR current sensor is to zero, the closer the measurement value is to the true value, i.e., the more accurate the sensor. As both the Hall current sensor and the TMR current sensor measure the magnetic field generated by the current indirectly, according to the standard for the accuracy grade of Hall current sensors [18,19], the TMR current sensor can reach an accuracy grade of 0.05. This shows that the TMR current sensor has very good accuracy.

### 4.2. Sensor Dynamic Characteristics Experiment

**Figure 10:**
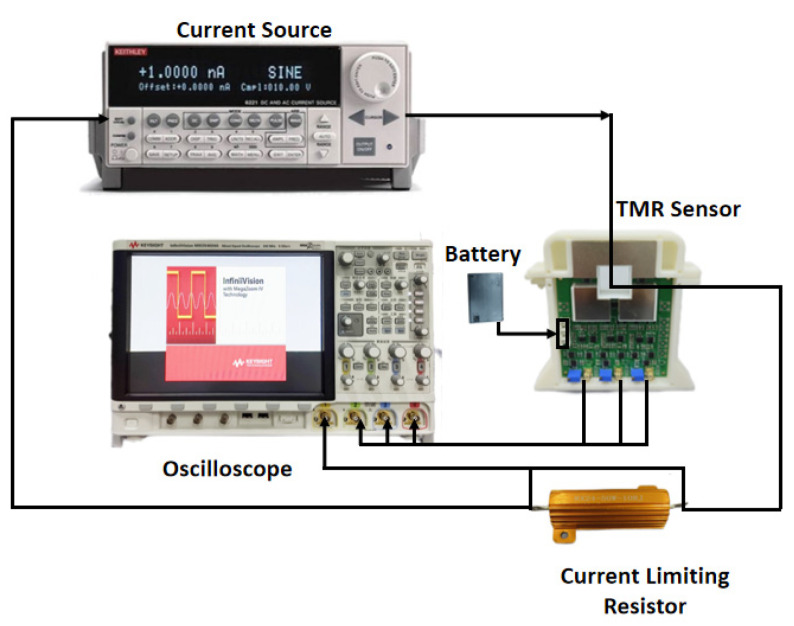
Frequency response test system.

When testing the frequency response of the sensor, the TMR current sensor is still placed in the shielding tube. The current source is provided by the Keithley 6221 from Tektronix Inc. A current-limiting resistor is connected in series with the load circuit. Channel 1 of the MSOX4024A oscilloscope is connected to the voltage across the current-limiting resistor, and the other channels are connected to the output voltage of the TMR current sensor. The oscilloscope is the frequency response test system, as shown in Figure 10.

**Figure 11:**
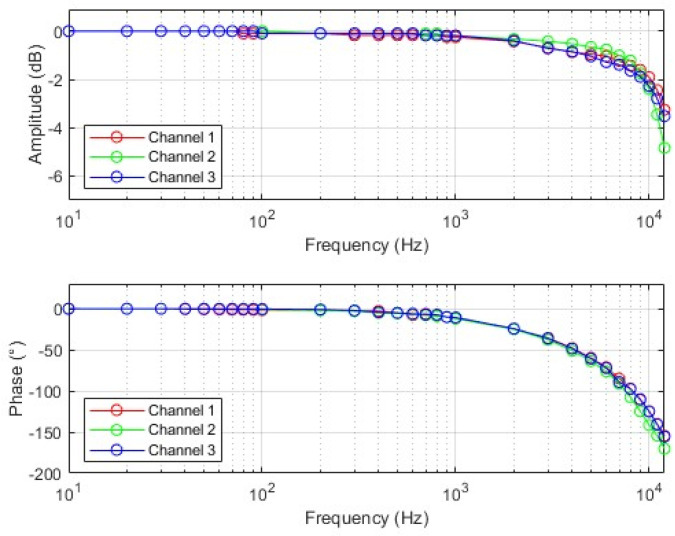
Sensor amplitude–frequency and phase–frequency characteristic test results.

The frequency response of the TMR current sensor refers to the amplitude–frequency characteristic and phase–frequency characteristic of the sensor. Within the set range of sinusoidal changing input current frequency, the amplitude of the sensor output voltage varies with frequency, and the phase shift between the sensor output voltage and the input current varies with frequency. The −3 dB bandwidth refers to the frequency range corresponding to the −3 dB drop in the amplitude of the current sensor output signal. The wider this bandwidth, the wider the frequency band that the sensor can measure. Figure 11 shows the results of amplitude–frequency and phase–frequency characteristic tests. The frequency response test shows that the sensor can achieve current measurement within a frequency of 10.5 kHz.

### 4.3. Sensor Resolution Experiment

**Figure 12:**
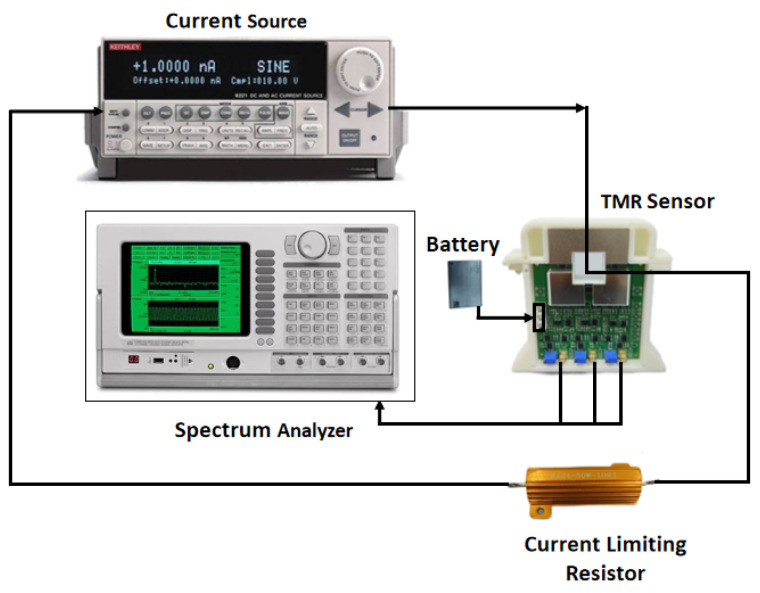
Voltage noise test system.

**Figure 13:**
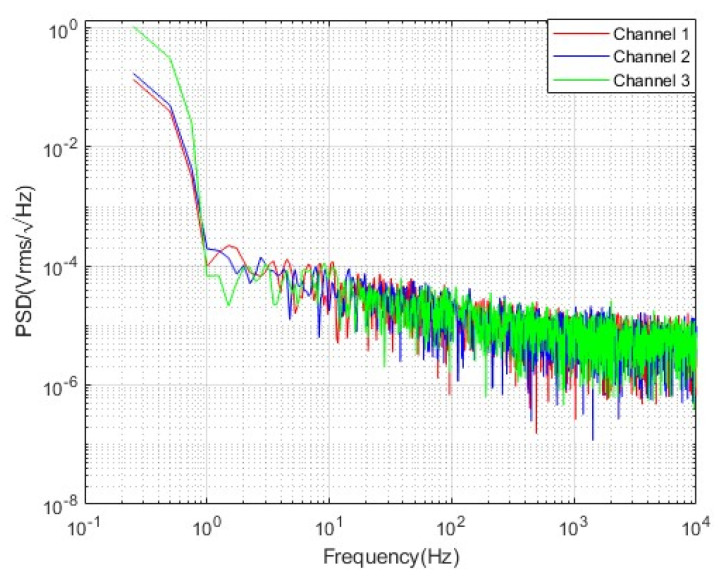
Current sensor voltage power spectral density.

Narrowband resolution refers to the smallest measurable current at a specific frequency, defined by the current noise power spectral density of the sensor at that frequency. To measure the resolution, the sensor’s background voltage noise power spectral density is first tested in a magnetically shielded environment with no current flowing through the conductor. The test system for voltage noise is shown in Figure 12. The current sensor’s voltage power spectral density is shown in Figure 13, with an average voltage noise of 20.27 μV/√Hz at 50 Hz. Using the sensor’s sensitivity at 1 V supply voltage, the narrowband resolution of the sensor was calculated to be 0.689 μA/√Hz at 50 Hz. Table 2 is a comparison of the performance parameters of the current sensor in this paper with other current sensors. The ETCR6300D is a commonly used current detection product. From Table 2, it can be seen that the current sensor designed in this paper has good parameter performance.

## 5. Conclusions

This paper presents the design and performance evaluation of a high-precision, high-resolution TMR current sensor tailored for weak current detection. The design process was detailed, encompassing experimental principle analysis, sensor structure design, chip selection, magnetic flux concentrator design, and the establishment of a corresponding test system. The proposed sensor demonstrates several key advantages, including a wide measurement range, high accuracy, high resolution, and non-invasive measurement capabilities. In terms of chip selection, this paper’s sensor design adopts a low-noise, low-hysteresis TMR chip; the sensor circuit uses a high-linearity interface circuit to eliminate fixed bias; and a magnetic flux concentrator is used to improve sensitivity and anti-interference capability. Experimental results indicate that the sensor achieves an average sensitivity of 29.4 mV/V/mA within a measurement range of ±100 mA. The sensor exhibits excellent linearity, with a deviation as low as 0.19%, and outstanding accuracy, with a deviation of only 0.045%. Furthermore, the sensor achieves a narrow-band resolution of 0.689 μA/√Hz at 50 Hz and is capable of performing current measurements from DC up to 10.5 kHz, with a phase shift of −145° at this frequency.

The sensor’s broad measurement range makes it particularly suitable for detecting weak currents, such as those associated with lightning arrester leakage currents and insulator leakage currents. Analysis of the sensor’s narrow-band resolution revealed that the primary factors influencing the minimum detectable current are the intrinsic noise of the sensor and its sensitivity. To further improve the detection limit, future work can focus on exploring new TMR film structures to enhance the sensitivity of the TMR chip, and optimizing the design of the sensor interface circuits based on actual problems to reduce the sensor’s noise. By addressing these two aspects, we expect to further improve the performance of the TMR-based sensor.

## Figures and Tables

**Table 1:** Performance comparison of magnetic sensors [10,11,12,13].

Performance	Hall	AMR	GMR	TMR
Power Consumption (mA)	5~10	1~10	1~10	0.001~0.1
Linearity Operating Range (Oe)	1~1000	0.001~10	0.1~100	0.001~1000
Sensitivity (mV/V/Oe)	~0.05	~1	~3	~100
Resolution (mOe)	~500	~0.1	~2	~0.1
Temperature Stability (°C)	<150	−40~+150	−40~+300	−40~+350

**Table 2:** Comparison of different current sensor parameters.

Parameter	ETCR6300D	[20]	[21]	This Paper
Sensitivity	/	10.52 mV/mA	30 mV/mA	≤33.9 mV/mA
Linearity	1.53%	0.12%	0.26%	≥0.06%
Accuracy	3.5%	/	0.24%	0.045%
Frequency range	/	10 KHz	10 KHz	10.5 KHz
Measured deviation	0.7 mA	/	0.486 mA	0.09 mA

## Data Availability

Experimental data can be obtained by contacting the author via email (xuyong22@mails.ucas.ac.cn).

## References

[1] Strasser T., Andrén F., Kathan J., Cecati C., Buccella C., Siano P. (2015). A Review of Architectures and Concepts for Intelligence in Future Electric Energy Systems. IEEE Trans. Ind. Electron..

[2] Zanella A., Bui N., Castellani P.A., Vangelista L., Zorzi M. (2014). Internet of Things for Smart Cities. IEEE Internet Things J..

[3] Chen Z., Chen X., Ding X., Wei L., Tang T., Zhou Y., Yu W. (2024). Electromagnetic interference compensation method of TMR current sensor. J. Phys. Conf. Ser..

[4] Wang S., Huang H., Yang Y., Chen Y., Fu Z., Jin Z., Shi Z., Xiong X., Zou X., Chen J. (2024). A Modulation Method for Tunnel Magnetoresistance Current Sensors Noise Suppression. Micromachines.

[5] Li D., Wang L., Jiao F., Peng G., Zhang S. (2022). Design and Application of Differential TMR Weak Current Sensor. Electr. Power Inf. Commun. Technol..

[6] Yuan X. (2021). Research on Detection Method of Leakage Current of Zinc Oxide Arrester. Ph.D. Thesis.

[7] Bai R., Li B., Tu J., Yang T., Dou A., Sun Y., Qian Z. (2024). Dual-range TMR current sensor based on magnetic shunt/aggregation effects utilizing single magnetic ring structure. Meas. Sci. Technol..

[8] Xu X.P., Liu T.Z., Zhu M., Wang J.G. (2020). New Small-Volume High-Precision TMR Busbar DC Current Sensor. IEEE Trans. Magn..

[9] Zhang P., Li Q., Zhang W., Liu C. (2019). Study on design of tunneling magnetoresistance current sensor. Instrum. Tech. Sens..

[10] Yang Q., Sun S., Sima W., He Y. (2019). Progress of Advanced Voltage/Current Sensing Techniques for Smart Grid. High Volt. Technol..

[11] Yan S.H., Zhou Z.T., Yang Y.D., Leng Q.W., Zhao W.S. (2022). Developments and Applications of Tunneling Magnetoresistance Sensors. Tsinghua Sci. Technol..

[12] Jiang Q. (2022). Design of Magnetic Sensor Based on AMR Effect and the Interface Circuit. Ph.D. Thesis.

[13] Lv H., Liu M.F., Cao W., Bai M., Wei F.L., Yang Z., Wang G. (2012). Performance and application of magnetic sensors based on TMR effect. J. Magn. Mater. Devices.

[14] Zhou Z., Yan S., Zhao W., Leng Q. (2022). Research progress of tunneling magnetoresistance sensor. Acta Phys. Sin..

[15] Xie B., Zhang G., Qin Y., Chen C. (2023). Micro grid overload current test method based on TMR sensor array. J. South-Cent. Univ. Natl. (Nat. Sci. Ed).

[16] Yu J., Long Z., Liang S., Yue C., Yin X., Zhou F. (2023). Optimal design of dual air-gap closed-loop TMR current sensor based on minimum magnetic field uniformity coefficient. Sci. Rep..

[17] Zhou L. (2019). Study on Current Sensing Technology Based on Tunnel Magnetoresistance Effect. Ph.D. Thesis.

[18] Hu J., Zhao S., Ouyang Y., He J., Wang S., Chang W. (2017). High performance current sensors based on giant magnetoresistance effect and practical applications in smart grids. High Volt. Eng..

[19] Li P., Yuan Z., Tian B., Li L., Yu L., Wang Z., Xu A., Xu Q., Lin Y., Shi X. (2019). Micro current measurement technology based on tunnel magnetoresistance. South. Power Syst. Technol..

[20] Hu J., Wang B., Sheng X., Zhao G., Zhao S., He J. (2020). Design and Noise Analysis of Weak Current Sensor with Broadband Based on Tunneling Magnetoresistance Effect. High Volt. Eng..

[21] Yang Y. (2024). Research on Key Technologies of High-Precision TMR Weak Current Sensor. Ph.D. Thesis.

